# Cognitive load impairs athletes' subliminal processing: behavioral and neural insights

**DOI:** 10.3389/fpsyg.2025.1704480

**Published:** 2026-01-27

**Authors:** Xuechen Mao, Jinbin Chen, Yanglan Yu, Qin Huang, Jilong Shi

**Affiliations:** 1 Department of Physical Education, Nanjing University of Chinese Medicine, Nanjing, China; 2 Department of Encephalopathy, Nanjing Hospital of Chinese Medicine Affiliated to Nanjing University of Chinese Medicine, Nanjing, China; 3 School of Psychology, Shanghai University of Sport, Shanghai, China; 4 Department of Physical Education, Leshan Normal University, Leshan, China; 5 Department of Physical Education, Xiamen University, Xiamen, China

**Keywords:** cognitive load, dual-task paradigm, event-related potential, subliminal processing, table tennis athletes

## Abstract

**Background:**

Athletes who play interactive sports are required to effectively process information under high cognitive loads. A growing body of research has shown that cognitive loading impairs athletes' supraliminal information processing, but few studies have focused on effects on subliminal processing. This study aimed to test the hypothesis that an increase in cognitive load impairs athletes' subliminal processing and its neural characteristics.

**Methods:**

Thirty national-level table tennis athletes (15 males; mean age 20.47 years) performed a dual-task paradigm (experimental), in which an N-back task was combined with a masked priming task. Subliminal priming effects were calculated using behavioral and electroencephalographic data to reflect subliminal processing levels.

**Results:**

The behavioral observations showed a significant decrease in the subliminal priming effect (i.e., response time) with increasing memory load (*p* = 0.001, $\eta_{\text{p}}^{2}$ = 0.40). The electrophysiological results showed significant decreases in the subliminal priming effect for the P3 amplitude (*p* = 0.01, $\eta_{\text{p}}^{2}$ = 0.26) and the pre-stimulus alpha power (*p* < 0.01, $\eta_{\text{p}}^{2}$ = 0.26) with increasing memory load, and a negative correlation between differences in these parameters under different load conditions (*p* < 0.01).

**Conclusions:**

The study findings suggest that the cognitive load impairs athletes' subliminal processing by consuming the available resource capacity. This study, performed with athletes with strong subliminal processing abilities, not only highlights the importance of athletes incorporating cognitive load into their daily training to enhance their competitive performance (e.g., adding memory tasks during sports training), but also deepens our understanding of the relationship between attention resources and subliminal processing. Additionally, the small sample size and the restriction to national-level table tennis players, both of which significantly constrain the generalizability of the results.

## 1 Introduction

With the vigorous development of modern competitive sports, greater requirements are placed on athletes' motor skills and cognitive abilities. Although athletes' cognitive processing characteristics have been explored extensively (Wang et al., 2025; Zhao et al., 2025), single-task paradigms focusing on specific information-processing processes have been adopted in most studies. In contrast, athletes, especially those playing open-skill ball sports, are required not only to effectively maximize the collection, retention and processing of information in complex and dynamic scenarios for tactical analysis and decision-making, but also to make rapid and accurate judgments about incoming balls (Fuster et al., 2025; Klotzbier and Schott, 2024). Thus, they must have good multitasking parallel information-processing abilities, i.e., they must process information effectively under heavy cognitive loads. However, the impact of the cognitive load on athletes' information processing has received limited attention in competitive sports research and practice.

The dual-task paradigm enables the exploration of this impact. Under experimental conditions, athletes are asked to simultaneously perform cognitive load and perceptual decision-making tasks for the investigation of effects on information processing under different cognitive loads. In one study, table tennis players were asked to return balls deployed from a ball machine while concurrently performing an auditory N-back task, and performance reductions were observed under high loads (Schaefer and Scornaienchi, 2020). Similarly, in another study, football players were required to make judgments about their teammates‘ kicking directions while performing the digital N-back task; when the N-back load increased, their perceptual task accuracy decreased and their reaction times (RTs) increased significantly (Yin et al., 2025). These consistent findings suggest that cognitive loads impair athletes' information processing. Notably, these studies share a common characteristic: all of the information-processing materials were presented supraliminally, without involving the subliminal level.

Distinct from conscious and preconscious processing, subliminal processing is applied to information presented below the threshold of conscious awareness (Dehaene et al., 2006; Mudrik and Deouell, 2022). In competitive sports such as table tennis, the presentation of motion information is extremely rapid, occurring below human awareness thresholds (Zagatto et al., 2018; Zou et al., 2024). One meta-analysis further showed that subliminal processing ability is more crucial for athletes competitive performance than is supraliminal processing ability (Jiang et al., 2021). Although some researchers have shifted their focus from the supraliminal to the subliminal information processing of athletes (Jiang et al., 2024; Shi et al., 2022), they have not integrated cognitive loading into their work, although information processing under cognitive loads more closely resembles real competitive situations (Farvardin et al., 2022; Scharfen and Memmert, 2019). Thus, cognitive loading must be considered when exploring the subliminal information processing of interactive sports players.

Cognitive neuroscience techniques enable the elucidation of the neural mechanisms underlying the influence of cognitive loading on athletes' subliminal processing. To simulate such processing in sports scenarios, subliminal stimuli are presented extremely briefly. In some studies, the presentation time of subliminal stimuli is 33 ms (Meng et al., 2019; Geng et al., 2020); while in other studies, the presentation time of subliminal stimuli is only 17 ms (Güldenpenning et al., 2015; Shi et al., 2024a; You et al., 2018). Electroencephalography (EEG), which can effectively detect neural activities induced by subliminal stimuli within tens of milliseconds, is an ideal method for this kind of study. In one study performed with table tennis players, diamonds and squares were used as subliminal priming stimuli in a masked go/no-go task with event-related potential (ERP) detection, and significant subliminal no-go P3 effects (larger P3 amplitudes under the no-go than under the go condition) were detected in the frontoparietal region (You et al., 2018). Another study involving a backward masked priming task with arrows serving as stimuli revealed significant P3 subliminal priming effects (SPEs) in the centroparietal regions of table tennis players (Meng et al., 2025). The P3 amplitude in the frontoparietal region has the same source as the no-go P3 amplitude (Polich, 2007), and is associated with inhibitory control processes (Azizian et al., 2006), suggesting that subliminal processing involves the participation of higher-level cognitive resources such as executive attention (Ansorge et al., 2014; Mao and Li, 2022a; Mao et al., 2022b). In other words, the mechanism of the cognitive load's influence on the subliminal processing of table tennis may be related to the allocation of cognitive resources; this speculation, however, needs to be confirmed with the support of empirical data. Moreover, pre-stimulus alpha power in the parieto-occipital area plays an important role in allocating attention and inhibiting irrelevant regions before stimulus presentation. The more resources available for processing stimuli, the stronger the pre-stimulus alpha power. Therefore, by integrating P3 amplitude and pre-stimulus alpha power, the mechanism underlying the impact of cognitive load on subliminal processing can be clarified. Previous studies have shown that table tennis players have advantages in subliminal processing (Geng et al., 2020; Jiang et al., 2024; Meng et al., 2019, 2025; 12-14, You et al., 2018), which they can perform with reduced resource expenditure (Shi et al., 2024b). Thus, the resource-demanding characteristics of subliminal processing could be better demonstrated by selecting table tennis players as study participants, and applying the theory of limited attentional resources to the field of unconscious processing.

In summary, although table tennis players, as representatives of individuals with typical subliminal processing abilities in sports, have been found in numerous studies to possess advantages in subliminal processing and even exhibit neural efficiency, it remains unknown whether this advantage is influenced by cognitive load. Yet, this scenario (i.e., under cognitive load) is much closer to the real-life context where table tennis players demonstrate their actual subliminal processing capabilities. Furthermore, the elucidation of the underlying brain mechanisms could improve our understanding of competitive athletes' information-processing characteristics under high time pressure and the relationships between cognitive resources and subliminal processing. Thus, this study aims to explore the effect of cognitive load on athletes' subliminal processing and its neural characteristics. We tested two hypotheses: (1) that increased cognitive loading would impair the athletes' subliminal processing (which requires attention resources) and (2) that this impairment would be due to the consumption of available attention resources in the frontoparietal region. Therefore, in this study we asked table tennis athletes to perform a dual-task paradigm (N-back and forward-backward masked priming), and used EEG and ERP measurements to explore how cognitive loads affected their subliminal processing.

## 2 Materials and methods

### 2.1 Participants

Based on a power analysis (G^*^Power 3.1, α = 0.05, power = 0.80, effect size = 0.25) (Faul et al., 2007), a recent meta-analysis (Kalén et al., 2021), and previous research including those employing the dual-task paradigm with subliminal priming tasks (Mao and Li, 2022a; Mao et al., 2022b, 2025) and those using EEG to examine table tennis athletes in subliminal priming tasks (Shi et al., 2024a; You et al., 2018), we determined that a minimum of 24 study participants was needed. To enhance statistical robustness, 30 table tennis athletes (15 males) were recruited from Shanghai Sports University and the Nanjing Sport Institute. The participants had an average age of 20.47 ± 0.33 years and an average of 10.21 ± 2.32 years of training experience. They trained three to five times per week, with each session lasting 2–5 h. The recruited athletes had achieved at least the second level in China's national ranking system. Considering that this experiment involved a dual-hand response task and the stimulus materials were not related to expertise, task performance was less affected by handedness and both left-handed (*n* = 5) and right-handed individuals were included. All participants were in good health and reported normal or corrected-to-normal vision with no color blindness or color weakness. The Ethics Committee of Shanghai University of Sport approved this study (No. 102772021RT020), which was conducted according to the ethical guidelines of the Declaration of Helsinki. All participants provided written informed consent and received compensation after completing the experiment.

### 2.2 Stimuli and apparatus

Ten Arabic numbers (0-9; horizontal × vertical, 0.7° × 1.0°) were used as stimuli for the working memory tasks. Five ellipses (4.0° × 1.1°-4.0° × 1.9°) and five diamonds (1.1° × 4.0°-1.9° × 4.0°) were adopted as stimuli for the subliminal priming tasks (Mao and Li, 2022a; Mao et al., 2022b). One ellipse (4.0° x 1.5°) and one diamond (1.5° x 4.0°) served as targets, and the remaining shapes served as primes. Forward and backward masks (4.0° x 4.0°) were drawn with many randomly oriented characteristics, and white crosses (1° x 1°) and yellow crosses (1.5° x 1.5°) served as fixation points. Altogether, 8 primes, 2 targets, 2 masks, and 10 memory items were white and were presented centrally on a 19-inch dark-gray (RGB: 128, 128, 128) screen at a viewing distance of approximately 70 cm.

The experiment was conducted using a computer monitor (resolution = 1400 × 900 pixels, frequency = 60 Hz, and frame duration = 16.67 ms) and the E-prime 2.0 software package. Brain electrical activity was detected using a 64-channel actiChamp system (Brain Products Inc., Gilching, Germany) with continuous recording at a sampling rate of 1,000 Hz and band-pass filtering at 0.01–100 Hz. It should be pointed out that all white stimuli were presented on the same gray background (RGB: 128, 128, 128) to ensure consistency in luminance and contrast levels.

### 2.3 Design and procedures

#### 2.3.1 Dual-task paradigm

**Figure 1:**
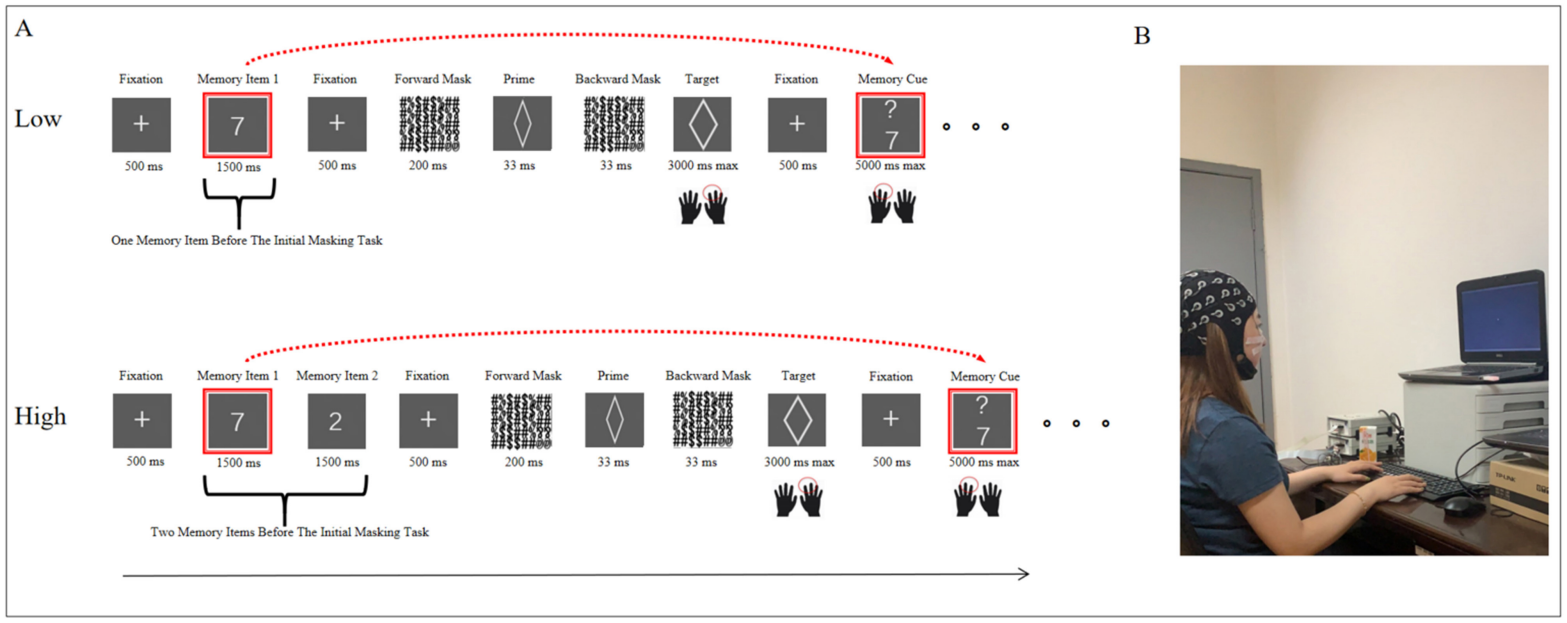
**(A)** Schematic illustration of the dual-task paradigm. **(B)** experience setup.

In each trial, the participant viewed a central yellow fixation point for 500 ms and then one memory item for 1,500 ms under the low-load condition; under the high-load condition, two memory items were displayed sequentially for 1,500 ms each (total, 3000 ms; Figures 1A, B). The participant was then asked to determine whether a newly displayed item was identical to the memory item (the first of these items under the high-load condition) after completing one subliminal priming task. Thereafter, a central white fixation point was shown for 500 ms and sequentially displaced by a forward mask (200 ms), a prime (33 ms), and a backward mask (33 ms). Next, a target was presented, and the participant was asked to indicate its shape with their right hand within 3,000 ms (right arrow for an ellipse and left arrow for a diamond). When a response was given or 3,000 ms had passed, a white fixation point was shown again for 500 ms, followed by a new memory item. This time, the participant was asked to indicate with their left hand whether the new item matched the one from the previous masking task (the first ten under the high load condition). The participant had to not only respond regarding the new memory item (“z” for “identical” “c” for “different”) but also retain the new memory item for comparison with the subsequent memory item presented after the completion of the next masking task (after the completion of two priming tasks under the high-load condition). When a response was given or after 5,000 ms had passed, the screen went blank and the next masking task started after 1,000 ms.

The main task was delivered before participants had practiced 60 shape discrimination tasks, 60 N-back tasks (30 under each loading condition), and 6 dual-task trials (3 under each loading condition). In the main task, 10 N-back tasks were intermixed with 10 masking tasks in one trial, and participants were asked to respond as accurately and quickly as possible. A total of 40 dual-task trials was administered under the two loading conditions, including 200 N-back tasks and 200 masking tasks under each condition. The two loading conditions were administered in turn; with the low-load and high-load conditions each administered first in half of cases. In the N-back task, half of the new items matched the old item and the other half did not. For the masking task, half of the trials were congruent and the other half were incongruent. Accordingly, 10 memory items, 2 targets and 8 primes appeared equally often. In this way, all correct key-press combinations had an equal probability of occurrence, so as to reduce participants' ability to predict the stimuli or key-press combinations.

#### 2.3.2 Visibility test

To ensure that the masked prime processing was subliminal, the participants completed a visibility test after the main task. This visibility test included objective and subjective measures to enhance its validity (Ansorge et al., 2014; Azizian et al., 2006; Mao and Li, 2022a; Polich, 2007). The procedure for objective measurement was identical to the masking task except that the shape discrimination was for the prime. Subsequently, the participants subjectively rated the visibility of the prime using a 4-point perceptual awareness scale (1, no experience; 2, weak glimpse; 3, almost clear; 4, absolutely clear). The visibility test was performed under the low-load condition as the suppression method used is more effective under high loads than under low loads (Mao et al., 2022b; Mao and Li, 2022a; Todd et al., 2005), and the participants were required to give responses as correctly as possible without time limit. Participants had to undergo 20 practice trials before initiating the identification task, which consisted of 100 identification trials, which contained 100 memory tasks and 100 subjective and 100 objective tasks. The participants completed the task carefully and thoroughly with unlimited time, ensuring accuracy as the priority.

### 2.4 EEG recording and pre-processing

EEG data recording was referenced online against the FCz site, and electrode impedances were kept below 5 kΩ. After recording, the EEGlab 13 software in MATLAB (version R2013a) was used for offline data preprocessing and analyses. The average potential of the mastoid electrodes (TP9 and TP10) served as the new reference. The EEG data were high-pass filtered at 0.1 Hz and low-pass filtered at 40 Hz (slope, 24 dB/octave). In addition, notch filters were applied at 50 Hz to eliminate noise. Ocular and any other residual artifacts were corrected through independent component analysis and visual inspection to ensure data quality. The quality of the data was high, with a mean of 2.42 ± 0.12 independent components removed during ICA preprocessing; these components primarily corresponded to vertical electrooculography and horizontal electrooculography. Epochs were then segmented from 447 ms before to 800 ms after target stimulus display onset. Given the masked priming paradigm employed in this study (please see Section 2.3.1), three stimuli, especially, a forward mask (200 ms), a masked prime (33 ms), and a backward mask (33 ms), were presented sequentially prior to the target, with a total duration of 266 ms. Consequently, the conventional baseline correction window (a 200-ms interval preceding the target) lack stability and freedom from contamination in the current study. The adoption of this conventional window would therefore introduce unintended biases to the results (Martens et al., 2011; Meng et al., 2025; Ortells et al., 2016; Shi et al., 2024a). Epochs with amplitudes exceeding ± 100 μV or trials with response errors were excluded. After these exclusions, the per-participant average for each condition comprised at least 70 epochs. EEG waveforms were averaged for each condition.

Based on previous findings that P3 amplitudes are related to the SPE in table tennis athletes (Meng et al., 2025) and are maximal in the frontocentral area (You et al., 2018), data from three frontocentral electrodes pairs (left, FC3 and C3; middle, FCz and CZ; right, FC4 and C4) in the 350–550-ms interval were selected for the analysis of the mean of these amplitudes. The P3 time window and regions of interest were defined based on previous findings and visual inspection of the grand-average waveforms and topographic maps.

The alpha power (8–12 Hz), which has been found to be sensitive to the cognitive load and related to the redistribution of attentional resources (Lenartowicz et al., 2021; Sghirripa et al., 2021; Zhang et al., 2025), was measured. Before averaging for each condition, the alpha power from 200 to 0 ms before target stimulus display onset was calculated using the complex Morlet wavelet transform, where t is time, f is frequency and σ represents the width of each frequency band, which was set as 3–10 logarithmically spaced cycles to trade-off temporal and frequency resolution, and then taking the inverse fast Fourier transform. Power was defined as the modulus of the resulting complex signal Z(real (z(t))2 + imag (z(t))2), and then normalized using a baseline of 467–267 ms before target display onset, using a decibel (dB) transform. The baseline activity was taken as the average power at each frequency band, averaged across conditions (dB power = 10 × log 10(power/baseline)). Considering that the alpha power is distributed predominantly in posterior cortical areas (Jiang et al., 2018; Silverstein et al., 2015; Zhao et al., 2022), we estimated it across parieto-occipital midline electrodes (Cz, CPz and Pz).

### 2.5 Analyses

To limit the influence of incorrect and extreme values, incorrect and missed trials and RTs below or above two standard deviations of the individual mean were rejected as outliers. The mean RT for correct responses and the mean error rate (ER) were calculated for each participant and experimental condition. The magnitude of the SPE on the behavioral data was quantified by subtracting the RT in congruent trials from that in incongruent trials. The magnitude of the SPE on the ERP data was quantified by subtracting the P3 amplitude in congruent trials from that in incongruent trials.

The statistical analyses were conducted using SPSS 25.0 (IBM Corporation, Armonk, NY, USA). Greenhouse–Geisser correction was used to compensate for sphericity violations, and the *post-hoc* tests underwent Bonferroni correction as needed. Least-significant-difference tests were used for multiple comparisons, and a simple effects analysis was used to explore interaction effects. *P* values < 0.05 were considered to be significant. Descriptive statistics (meanS and standard errors) were calculated, and partial eta-squared ($\eta_{\text{p}}^{2}$) values were computed to determine the main and interaction effect sizes.

#### 2.5.1 Analysis of visibility test data

To assess objective prime detection, d' scores were used, hit (correct answers in congruent trials) and false-alarm (erroneous answers in incongruent trials) rates were calculated for each participant. One-sample *t* tests were used to assess differences between d' values and 0 and between objective ERs and the chance level of 50%. Additionally, a Pearson's correlation analysis was conducted to examine the relationships between SPE magnitudes and d' values. Data from participants with objective ERs significantly below the chance level were removed from the analysis because we could not determine whether these participants had seen the masked prime.

#### 2.5.2 Analysis of N-back task data

Data from participants with N-back task ERs significantly below 2 standard deviations of the group mean were removed because we could not confirm whether these participants understood the task instructions. Repeated-measures analyses of variance (ANOVAs) of mean RTs and ERs with the working-memory load (low vs. high) serving as a within-subject factors were performed.

#### 2.5.3 Analysis of subliminal priming task data

Assumptions for parametric tests (homoscedasticity, examined using Levene's test, and normality of distributions, examined using Kolmogorov–Smirnov test) were examined. Then, two-way repeated-measures ANOVAs of mean RTs, ERs and alpha powers with the working-memory load (low vs. high) and prime congruency (congruent vs. incongruent), and three-way repeated-measures ANOVAs of mean P3 amplitudes with the working-memory load (low vs. high), prime congruency (congruent vs. incongruent) and location (left vs. middle vs. right) were performed.

To examine whether the P3 amplitude was related to the SPE (represented by RTs across loads, Pearson's correlations between differences in the SPE magnitudes for P3 amplitudes and RTs under different load conditions were assessed. To further determine whether the pre-stimulus alpha power was related to P3 SPE overloads, the association between differences in the SPE magnitude for P3 and the alpha power under different load conditions was also examined.

## 3 Results

Six participants were excluded from the analysis due to the following reasons: (1) their ERs in the objective visibility test were significantly lower than the chance level (*n* = 2); (2) their ERs in the N-back task were significantly higher than the chance level (*n* = 2); and/or (3) the quality of their EEG data in the masked priming task was poor (*n* = 4), specifically, after artifact removal, the number of segmented data trials under a single condition was fewer than 40 (*n* = 2). The final sample size (*n* = 24 after exclusions) meets the requirements of statistical power (power = 0.82). The mean percentage of trials retained for after artifact rejection was 77.38 ± 0.88% for congruent-low condition, 77.50 ± 0.82% for incongruent-low condition, 76.21 ± 1.34% for congruent-high condition, 75.12 ± 1.31% for incongruent-high condition. In addition, there were not systematic differences in demographic characteristics, athletic experience, or task performance between included and excluded participants and the exclusion rate was comparable between the two load conditions.

### 3.1 Visibility test

No participant reported having seen the prime stimuli in the subjective threshold evaluation (Supplementary Figure 1). Objective measurement yielded an ER of 47.80 % (Supplementary Figure 2), which did not differ significantly from chance (*t*_(23)_= −1.19, *p* = 0.25). The d' value was −0.04 (95% CI [−0.18, 0.97]) and did not deviate significantly from 0 (*t*_(23)_ = −0.61,*p* = 0.55). The d', SPE, the difference of the P3 priming effect under different loads and the difference of Alpha power under different loads distributions were normal (K–S = 0.10, *p* = 0.20; K–S = 0.17, *p* = 0.07; K–S = 0.13, *p* = 0.20 and K–S = 0.12, *p* = 0.20, respectively), and the d' values did not correlate with SPE (r_(24)_ = 0.32, 95% CI [0.11, 0.75], *p* = 0.12), nor did it correlate with the difference in the P3 priming effect across different loads (r_(24)_ = 0.34, *p* = 0.10) or the difference in alpha power across different loads (r_(24)_ = −0.22, *p* = 0.313). These results suggest that masking was effective and prime stimulus processing was subliminal.

### 3.2 N-back task results

Cognitive load manipulation was efficacious. The load had a main effect on RTs (*F*_(1, 23)_ = 8.87, *p* = 0.007, $\eta_{\text{p}}^{2}$ = 0.28, 90% CI [0.05, 0.47]; 2.50% and 5.23% outliers under low and high loads, respectively). RTs were significantly shorter under the low load than under the high load, with a difference of 77.12 ± 25.89 ms. The load also had a main effect on RA (*F*_(1, 23)_ = 4.29, *p* = 0.05, $\eta_{\text{p}}^{2}$ = 0.16, 90% CI [0.00, 0.36]). ERs were consistently higher under the high load than under the low load, with an RA difference of 1.21 ± 0.60%. In addition, a Mixed ANOVA that included the handedness factor revealed a significant main effect of load for RT (*F*_(1, 22)_ = 5.26, *p* = 0.03, $\eta_{\text{p}}^{2}$ = 0.19), and no significant interaction between the dominant hand and the cognitive load conditions for either RT (*F*_(1, 22)_ = 1.96, *p* = 0.18, $\eta_{\text{p}}^{2}$ = 0.08) or RA (*F*_(1, 22)_ = 0.17, *p* = 0.68, $\eta_{\text{p}}^{2}$ < 0.01). These findings indicate that the handedness factor did not influence the results.

### 3.3 Subliminal priming task results

#### 3.3.1 Behavioral results

**Figure 2:**
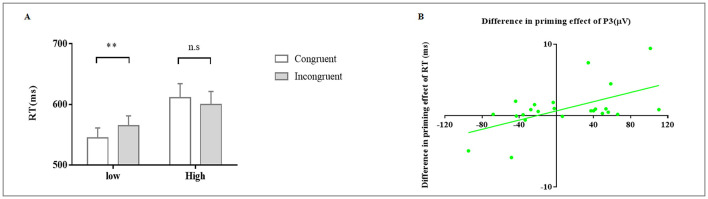
**(A)** RTs in the subliminal priming task, ** *p* = 0.01, n.s., no significance. **(B)** Relationship between the differences in the P300 and RT SPEs in the subliminal priming task under low- and high-load conditions

The load had a main effect on RTs (1.96% and 4.10% outliers under low and high loads, respectively; *F*_(1, 23)_ = 12.35, *p* = 0.002, $\eta_{\text{p}}^{2}$ = 0.35, 90% CI [0.10, 0.53]) (Table 1). RTs were significantly shorter under the low load than under the high load, with an average difference of 50.53 ± 14.38 ms, suggesting that the participants consumed more cognitive resources under the high-load condition. Significant interaction between the load and congruency was detected (*F*_(1, 23)_ = 15.36, *p* = 0.001, $\eta_{\text{p}}^{2}$ = 0.40, 90% CI [0.14, 0.57]). RTs were significantly smaller in congruent trials than in incongruent trials (*p* = 0.01) under the low load, but did not differ between congruent and incongruent trials under the high load (*p* = 0.12). These results suggest that the SPE decreased significantly with increasing cognitive load (Figure 2A). No significant main effect of congruency was found (F _(1, 23)_ = 0.65, *p* = 0.43, $\eta_{\text{p}}^{2}$ = 0.03), and no significant effect on ERs was identified.

**Table 1:** Mean reaction times (RTs in milliseconds) as a function of cognitive load (1-back vs. 2-back), and congruency conditions (congruent vs. incongruent) in the subliminal priming task.

		**Low**	**High**
Condition	Congruent condition	544.47 ± 16.59	610.75 ± 21.99
	Incongruent condition	564.64 ± 16.60	599.42 ± 20.59

RT, response time; RA, response accuracy. mean ± standard error.

For RT, a Mixed ANOVA incorporating the handedness factor revealed a significant main effect of load (*F*_(1, 22)_ = 5.64, *p* = 0.03, $\eta_{\text{p}}^{2}$ = 0.20), a significant two-way interaction between load and congruency (*F*_(1, 22)_ = 4.78, *p* = 0.04, $\eta_{\text{p}}^{2}$ = 0.18), and no significant interaction between dominant hand and load (*F*_(1, 22)_ = 1.71, *p* = 0.21, $\eta_{\text{p}}^{2}$ = 0.07), or between dominant hand and congruency (*F*_(1, 22)_ = 0.59, *p* = 0.45, $\eta_{\text{p}}^{2}$ = 0.03), or among dominant hand, load and congruency (*F*_(1, 22)_ = 1.33, *p* = 0.26, $\eta_{\text{p}}^{2}$ = 0.06). These findings indicate that the observed differences were not attributable to an imbalance in handedness. In addition, no significant effect on ERs was identified.

#### 3.3.2 Neurophysiological results

##### 3.3.2.1 P3 component

**Figure 3:**
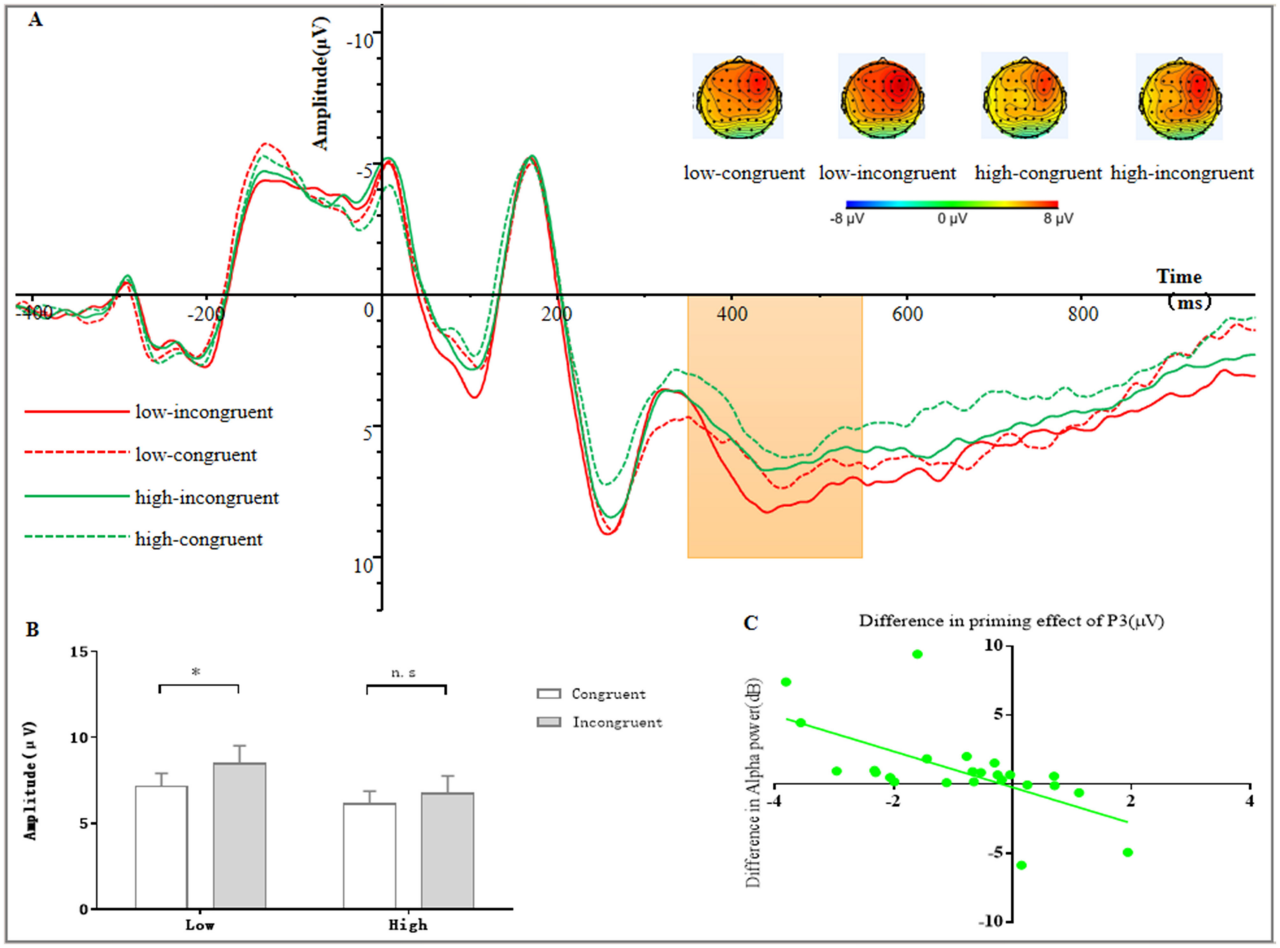
**(A)** Average amplitudes of the P3 components with topographic maps of the difference waves. **(B)** Statistical comparisons of mean amplitudes. *p < 0.05. **(C)** Relationship between the differences in the P300 SPE and pre-target alpha power in the subliminal priming task under low- and high-load conditions.

Three-way repeated measures ANOVA revealed a significant main effect of the load on the P3 amplitude (*F*_(1, 23)_ = 76.62, *p* < 0.001, $\eta_{\text{p}}^{2}$ = 0.77, 90% CI [0.59, 0.84]). Amplitudes were significantly higher under the low load than under the high load, with an average difference of 1.38 ± 0.16 μV. This result indicates that participants had more cognitive resources available to perform the subliminal priming task under the low-load condition. Interaction between the load and congruency was detected (*F*_(1, 23)_ = 7.95, *p* = 0.01, $\eta_{\text{p}}^{2}$ = 0.26, 90% CI [0.04, 0.46]). *Post-hoc* tests showed that the SPE for the P3 amplitude was significantly greater, reflecting more extensive processing of subliminal stimuli, under the low-load condition than under the high-load condition (1.30 ± 0.63 vs. 0.62 ± 0.55 μV). No other significant effect was observed. Waveform and topographic maps and mean P3 amplitudes for the subliminal priming task are shown in Figures 3A, 3B.

The difference in the magnitude of the SPE for the P3 amplitude under different load conditions (K–S = 0.08, *p* = 0.09) correlated positively (i.e., increased along) with the difference in the magnitude of the SPE for RTs under different load conditions (K–S = 0.14, *p* = 0.20; r_(24)_ = 0.57, *p* = 0.004; Figure 2B). Thus, changes in cognitive resource availability due to the difference in the load condition were reflected in corresponding changes in the SPE for the P3 amplitude, which might explain the significant difference in subliminal stimulus processing (i.e., RTs) between load conditions.

##### 3.3.2.2 Alpha band

The load had a significant main effect on the pre-stimulus alpha power (*F*_(1, 23)_ = 8.14, *p* = 0.009, $\eta_{\text{p}}^{2}$ = 0.26, 90% CI [0.04, 0.46]). This power was significantly stronger, suggesting enhanced desynchronization, under the low load than under the high load, with an average difference of 0.85 ± 0.30 dB.

The difference in the magnitude of the P3 SPE under different load conditions correlated negatively with the difference in the alpha power under different load conditions (K–S = 0.09, *p* = 0.20; r_(24)_ = −0.62, *p* = 0.001; Figure 3C). This relationship underscores the link between short-lasting changes in the brain's excitability state before subliminal stimulus processing, as reflected by the difference in the alpha-band magnitude under low and high loads, and changes in the availability of cognitive resources for subliminal priming task performance, as reflected by the difference in the magnitude of the P3 SPE under low and high loads.

## 4 Discussion

This study was performed to address a gap in the extant literature via the exploration of the effects of the cognitive load on table tennis athletes' subliminal perceptual processing using a dual-task (N-back and masked priming) paradigm and the detection of associated neural activities by EEG with ERP measurement. The results support the study hypotheses: the athletes' subliminal processing ability decreased with increasing cognitive load, in relation to changes in the amount of available cognitive resources in the frontoparietal cortex. These findings enhance our understanding of the subliminal information processing characteristics of fast-ball sports players and of the relationship of this processing to cognitive resources.

The behavioral results indicated that table tennis athletes showed good subliminal processing abilities under low cognitive loading, consistent with reports that athletes have superior unconscious executive control abilities (Huang et al., 2024; Meng et al., 2019; You et al., 2018). Executive control ability is vital to the performance of athletes engaged in interactive sports, as it enables athletes not only to suppress inappropriate action impulses but also to inhibit the cognitive interference caused by opponents' deceptive maneuvers (Raab et al., 2005). However, in competitive interactive sports, crucial movement information is presented subliminally due to the high speeds at which athletes and objects move (e.g., fast serves, rapid action sequences), which exceed the human perception threshold (Kibele, 2006). Thus, compared with conscious executive control, unconscious executive control better reflects the cognitive advantages of athletes (Jiang et al., 2021; Meng et al., 2019). Consequently, even under low cognitive loading, where a certain degree of resource depletion occurred, athletes could still utilize their remaining resources to conduct effective unconscious information processing in the dual-task paradigm.

Our finding that table tennis athletes' subliminal processing was reduced under a high cognitive load aligns with previous findings that the working-memory load interferes with athletes' behavioral performance (Farvardin et al., 2022; Fuster et al., 2025; Klotzbier and Schott, 2024; Schaefer and Scornaienchi, 2020; Yin et al., 2025). However, the use of a dual-task paradigm with subliminal stimulus presentation in the current study is a major difference from previous research. In previous research (Schaefer and Scornaienchi, 2020; Yin et al., 2025), the decision-making task stimuli that were affected by cognitive loading were presented at the supraliminal level (athletes were asked to respond to a stationary ball while performed a working-memory task), which is not completely consistent with real sports scenarios. Accordingly, the approach used in the current study has greater ecological validity than do those used in previous research. Moreover, it has not, to our knowledge, been used previously to explore the mechanisms of table tennis athletes' information processing under cognitive loading.

On the other hand, our behavioral results are inconsistent with the results of one study, in which a secondary cognitive load task did not weaken athletes' primary task performance (Praça et al., 2024). In that study, players were asked to play a three-a-side game for 4 mins under four conditions: control, with a secondary motor task, with an additional related secondary cognitive task and with an additional non-specific secondary task. Neither additional secondary cognitive load significantly impacted the athletes' physical performance. However, the participants showed different levels of engagement in the two secondary tasks, indicating that they shifted their attention to the primary task at the expense of secondary task performance. Thus, caution should be exercised when drawing conclusions from scenarios in which simultaneous active engagement in two tasks cannot be ensured. In the present study, cognitive load tasks were combined with subliminal processing tasks on a trial-by-trial basis, which may be an effective method to ensure simultaneous active engagement in both. In addtition, our dual-task deaign ensures cognitive load manipulation during the priming task, but it also means that the memory retrieval/comparison process occurs after subliminal stimulus processing. To limit the possibility that participants strategically reallocate attention between the two tasks in ways that could complicate interpretation, there is a significant difference in difficulty between the two tasks. Unlike the memory task, which requires updating, maintenance, and subsequent retrieval/comparison, the priming task is relatively straightforward—it involves making judgments about figures. To prevent participants from allocating excessive attentional resources to figure judgment, this task included only two target stimuli, both of which were simple (a diamond or ellipse). Additionally, during the pilot experiment, participants reported that figure judgment was almost completed automatically, requiring little attentional investment. They stated that they would direct more attention to the memory task, as it was more challenging.

Our observation of a significant decrease in the P3 amplitude in the frontocentral cortex with increasing cognitive load is consistent with previous findings showing that this amplitude is sensitive to the working-memory load (Lenartowicz et al., 2021; Vogel et al., 2005). Considering that the P3 amplitude is a recognized indicator of attention resource availability (Morrison et al., 2019; Ren et al., 2023; Scharinger et al., 2017; Wei and Zhou, 2020), this result suggests that our cognitive load manipulation was effectual and that the cognitive resources available for subliminal stimulus processing were depleted with increasing cognitive load. More importantly, the SPE of the P3 amplitude was significant under the low load, but weakened or even disappeared under the high load. Given that the magnitude of P3 subliminal priming is thought to index the subliminal stimulus processing level, these findings indicates that the athletes' subliminal processing was impaired due to the reduction of attention resources caused by the increased cognitive load. Table tennis athletes have been demonstrated to have excellent subliminal processing abilities (Jiang et al., 2021, 2024; Geng et al., 2020; Meng et al., 2025) and greater neural efficiency (i.e., less resource taxing for this processing) than do non-athletes (Guo et al., 2017; Huang et al., 2024; Shi et al., 2024b). Thus, the results of this study provide strong support for the view that subliminal priming generally requires resources, especially advanced cognitive resources (i.e., executive attention required by working memory). Our findings are in agreement with the existing literature, which extends the applicability of the resource limitation hypothesis from supraliminal to subliminal processing (Hung et al., 2020; Martens and Kiefer, 2009).

Notably, this work lends credence to the two-pool (working memory and subliminal priming) model of resource requirements (Mao et al., 2022b; Mao and Li, 2022a). According to that model, the retention and executive resource pools, which are relatively attention independent, are responsible for the maintenance and manipulation of working-memory subsystems, the latter of which shares the executive resource pool with subliminal priming. Previous studies have found that the subliminal priming effect does not decrease with increasing cognitive load by introducing maintenance load in the dual-task paradigm (Bodner and Stalinski, 2008; Perea et al., 2018). Current study found that the subliminal priming effect significantly decreased with increased cognitive load by introducing manipulation load in the dual-task paradigm. In those studies with increased maintenance load, executive attention is spared and is available to the masked prime; thus, subliminal priming is preserved. In the present study, the athletes' manipulation subsystems consumed large amounts of the attention resources in the executive resource pool when the cognitive load was increased by switching from a one-back to a two-back task, leaving insufficient resources for subliminal stimulus processing and thereby attenuating subliminal priming.

Although there are differences in their emphases, both the Global neuronal workspace theory (Dehaene et al., 1998; Dehaene and Naccache, 2001; Kouider and Dehaene, 2007) and the Recurrent Processing Theory (Lamme, 2010) hold that subliminal processing is essentially transient and of very limited complexity and thus does not require advanced cognitive resources such as frontoparietal attentional resources. Therefore, frontoparietal P3 component plays a key role in distinguishing between conscious and subliminal processing and reflects a neural marker of conscious processing (Boly et al., 2013; Dehaene and Changeux, 2011; Lamy et al., 2009). Hence, our ERP/alpha findings challenge these standard cognitive theories by revealing that subliminal processing can also induce the P3 priming effect, and this effect will be impaired by increased load in the manipulation subsystem. Our findings thus deepen the understanding of the relationship between the cognitive load and subliminal processing and support the view that subliminal processes are considerably more complex and durable than currently believed (Diao et al., 2021; Silverstein et al., 2015).

Our finding that a higher N-back load reduced the pre-stimulus alpha power in the parieto-occipital area is in line with previous findings (Bahrami et al., 2007; Ding et al., 2025; Kardan et al., 2020). Alpha oscillatory activity reflects alternating states of cortical inhibition and excitation, with higher alpha power in specific phases leading to more inhibition (Jensen and Mazaheri, 2010; Mathewson et al., 2011; Klimesch, 2012; Iemi et al., 2022). According to this view, alpha synchronization is characteristic of an inhibited brain state, whereas alpha, desynchronization is characteristic of an activated brain state. Therefore, the detection threshold for visual stimuli depends significantly on the pre-stimulus alpha power (Ergenoglu et al., 2004; Hanslmayr et al., 2005; van Dijk et al., 2008). This means that the lower the alpha desynchronization before stimulus presentation, the lower the ability to discriminate the stimulus. Consequentially, the current data on alpha power confirm that the low-load testing of d' was sufficient and the load manipulation was effective in this study because participants' visual cortical excitability significantly decreased with an increased cognitive load. Most importantly, the differences in alpha power under different load conditions correlated negatively with the difference in the P3 SPE magnitude, consistent with previous findings indicating that the pre-stimulus alpha power negatively predicts subliminal P3 effects (Ergenoglu et al., 2004; Silverstein et al., 2015) and is linked to mechanisms that modulate subliminal processing (Menétrey et al., 2023). Our findings support the pypothesis that enhancement of rhythmic activity in the alpha frequency band prior to the presentation of a stimulus may subserve as a gating mechanism for incoming sensory information on its way to the cortex (Ergenoglu et al., 2004; Hanslmayr et al., 2005; Stam et al., 1999; van Dijk et al., 2008) and suggest that participants' cortical arousal level decreased due to an increment in the cognitive load, and their subliminal processing weakened due to reduced resource availability. Thus, our EEG/ERP observations not only reveal the mechanism by which the cognitive load affects subliminal processing by regulating the available resource capacity, but also provide evidence that the alpha rhythm and P3 activity are associated with the mechanism by which the cognitive load regulates unconscious processing.

Our results that the table tennis athletes' subliminal processing was impaired by cognitive loading emphasizes the importance of athletes incorporating cognitive load into their daily training to enhance their competitive performance. Accumulating evidence demonstrates that modern competitive athletes are required to process subliminal information while coping with high time pressure (Meng et al., 2019; Shi et al., 2024a; You et al., 2018). While doing so, athletes in complex and dynamic open-skill sports scenarios must simultaneously effectively and maximally collect, memorize, and process information (i.e., conduct technical and tactical analyses and make decisions). Thus, cognitive loading should also be considered when designing athletes‘ daily training routines. The paradigm adopted in the present study provides insights into unconscious processing training for athletes under cognitive load. For example, athletes can perform memory updating tasks—such as mental arithmetic like sequentially adding 3 to numbers—while practicing hitting, to increase cognitive load. Cognitive load should be increased gradually. For instance, one could start with low load and then progress to high load; alternatively, an attempt could be made to begin with tasks related to the specific sport discipline, followed by tasks unrelated to it. Of course, all these approaches need to be based on more extensive applied research. Therefore, this study only provides theoretical suggestions, and more specific operations should be designed in conjunction with different sports and tailored to specific contexts.

Although the current work constitutes pioneering research on the effects of the cognitive load on subliminal processing in table tennis athletes, it has potential methodological limitations. First, the dual-task paradigm employed in this study consisted of N-back and masked priming tasks. Although forward and backward masking is effective for subliminal stimulus processing, other masking methods (e.g., continuous flashing suppression) could be applied in future research to confirm the generalisability of the present observations. Second, general geometric shapes were used as the masked primes and targets in this study. As the association of subliminal stimuli with sports scenarios may affect athletes' subliminal processing performance under cognitive loading (Kalén et al., 2021). sport-specific stimuli (e.g., table tennis ball, racket, service actions) should be used for load tasks or subliminal processing tasks in future studies to improve our understanding of the relationship between the cognitive load and table tennis athletes' subliminal processing in real sporting contexts. For example, when using table tennis stimuli or rally scenarios, athletes' subliminal processing performance may be less affected by cognitive load and may also exhibit better performance than that with general stimuli. This study indicates that athletes' ability of subliminal processing cannot be free from the demand for cognitive resources, at least under the condition of irrelevant stimuli. However, it is worth further exploring in future studies whether athletes can achieve better performance under cognitive load in sports contexts. Third, this study relied heavily on electrophysiological measurements, which, although informative, do not capture the full spectrum of neural activity. The exclusive focus on P3 and alpha activity may have led to the overlooking of other signals linked to the mechanisms by which the cognitive load affects subliminal processing, although this activity has been confirmed to be linked to these mechanisms (Meng et al., 2025; Shi et al., 2024a) and significant associations of P3 activity with subliminal processing and the alpha power were found in this work (Bazan, 2017; Cohen and Donner, 2013). Subsequent research endeavors could include additional subliminal processing-related potentials, such as the N2 component (van Gaal et al., 2011), and the exploration of other frequency bands, such as the theta band (Diao et al., 2021), for comprehensive analysis. Additionally, although the present study adopted previous research methods and also observed similar results—specifically, the negative correlation between alpha power differences and P3 SPE differences (Ergenoglu et al., 2004; Silverstein et al., 2015)—we acknowledge that this cannot rule out the existence of other possibilities. For instance, it is plausible that these two indices independently reflect cognitive load without a direct association between them. Therefore, further research in the future is necessary, particularly by employing more detection methods and analytical approaches (e.g., trial-by-trial alpha-ERP relationships, source localization) to further validate and enrich the findings of this study.

Furthermore, this study was conducted with a relatively small sample and included only table tennis athletes. The small sample size and the restriction to national-level table tennis players, both of which significantly constrain the generalizability of the results. Additionally, the absence of such comparison limits conclusions about the specificity of these findings to athletic populations. Although existing studies have identified the restrictive effect of cognitive load on subliminal processing by including non-athlete participants (Mao et al., 2022b; Mao and Li, 2022a), the present study needs to add non-athletes as a control group to more fully demonstrate that this restrictive effect is universal rather than specific to a particular group. Future investigations could also recruit more participants and incorporate control groups of athletes from different sports to compare and substantiate the results of this work. Moreover, While not the focus of this study, it should be noted that the dual-task paradigm employed herein cannot effectively distinguish the distinct roles of different resource pools in subliminal processing. To properly test the “two-pool model,” future investigation should design conditions that separately vary maintenance vs. manipulation demands. Finally, although the analysis pipeline utilized in this study is based on existing EEG/ERP studies on subliminal processing (Kiefer and Martens, 2010; Martens et al., 2011; Meng et al., 2025; Shi et al., 2024a), integration with the following community-validated tools represents a good opportunity for enhanced reproducibility. The BEST (Brain Electrophysiological recording & STimulation) Toolbox provides comprehensive guidelines and standardized protocols for EEG/ERP data collection, preprocessing, and analysis that could have strengthened this work (Hassan et al., 2022). Similarly, standardized pipelines such as EEGLAB's PREP pipeline for artifact correction (Bigdely-Shamlo et al., 2015) and the BIDS-EEG data structure for organizing and sharing electrophysiological data (Pernet et al., 2019) represent best practices that facilitate transparency and replication. The ERPLAB Toolbox also provides validated, standardized approaches for ERP component quantification that could enhance methodological rigor (Lopez-Calderon and Luck, 2014). Future investigations in this domain can consider adopt such standardized frameworks to not only facilitate direct comparison across studies examining cognitive load effects on subliminal processing, but also promote open science practices through improved data sharing and methodological transparency. Given the potential translational implications for athletic training, establishing reproducible protocols is particularly important for enabling validation and extension of these findings by other research groups.

## 5 Conclusions

The present study was conducted to assess the effects of the cognitive load on the subliminal processing of table tennis athletes, who are known to have advantages in such processing, using a novel dual-task paradigm. A pronounced reduction in the SPE with increased cognitive loading was observed at the behavioral and electrophysiological levels. With an increased cognitive load, executive attention resources were consumed, leading to a decrease in subsequent cortical activation. As a result, significantly fewer resources were available for subliminal stimulus processing, impairing this processing. Stimulus presentation is typically subliminal in open-skill sports, and our observations effectively characterize table tennis athletes' information processing in sports scenarios. The dual-task paradigm used is not only a valuable framework for the further investigation of information processing mechanisms among open-skill sports players but also a practical strategy for the improvement of athletes' performance under time pressure. Furthermore, the EEG/ERP analyses performed in this work elucidated the relationship between attention resources and subliminal processing. This not only extends the applicability of the resource limitation hypothesis beyond the supraliminal level but also demonstrates that the resources required for subliminal processing are higher-order in nature; specifically, frontoparietal attentional resources.

## Data Availability

The raw data supporting the conclusions of this article will be made available by the authors, without undue reservation.
